# Pattern Recognition by Melanoma Differentiation-Associated Gene 5 (Mda5) in Teleost Fish: A Review

**DOI:** 10.3389/fimmu.2019.00906

**Published:** 2019-04-26

**Authors:** Jassy Mary S. Lazarte, Kim D. Thompson, Tae Sung Jung

**Affiliations:** ^1^Laboratory of Aquatic Animal Diseases, College of Veterinary Medicine, Gyeongsang National University, Jinju, South Korea; ^2^Moredun Research Institute, Pentlands Science Park, Penicuik, United Kingdom

**Keywords:** teleost fish, innate immune system, pattern recognition receptors, melanoma differentiation-associated gene 5, interferon pathway

## Abstract

Teleost fish, as with other vertebrates, rely on their innate immune system as a first line of defense against invading pathogens. A very important characteristic of the innate immune response is its ability to recognize conserved molecular structures, such as viral dsRNA and ssRNA. Mda5 is one of the three pattern recognition receptors (PRRs) that recognize cytoplasmic viral ligands. Teleost Mda5 is widely conserved among several fish species and possesses the same structural domains as those seen in their mammalian counterparts. Fish Mda5 has been shown to be capable of initiating an inflammatory response both *in vitro* (in different fish cell lines) and *in vivo* using synthetic viral analogs or virus. The interferon (IFN) pathway is triggered as a result of Mda5 activation, leading to the expression of type I IFNs, IFN- stimulated genes and pro-inflammatory cytokines. Although it is known that Mda5 acts as a receptor for virally-produced ligands, it has been shown more recently that it can also initiate an immune response against bacterial challenges. This review discusses recent advances in the characterization of teleost Mda5 and its potential role in antiviral and antibacterial immunity in teleost fish.

## Introduction

Vertebrates have both innate and adaptive immune systems that help them defend themselves against pathogens, such as viruses and bacteria. The innate immune system acts as the initial line of defense against infection and plays a pivotal role in mediating an immediate immune response, which in turn helps to activate the adaptive immune system (1). While higher vertebrates (i.e., mammals), have a much more complex adaptive immune system compared to lower vertebrates, studies have shown that lower vertebrates (including fish) have an intricate innate immune system that compensates for their less developed adaptive immune system (2).

Early pathogen recognition is paramount for an organism's survival. It is important that the host has a set of “sensors” that can instantly recognize the presence of microbial/viral nucleic acids within its cytoplasm. One of these is the DExD/H-box (DDX) protein family that includes RNA and DNA helicases possessing a DExD/H-box domain. DDX proteins are directly involved in the regulation of gene induction and other important processes including signal transduction, gene promoter regulation, mRNA splicing, translational regulation and most importantly, they have been implicated in innate immunity, acting as RNA sensors or signaling molecules (3). Another group of receptors that recognize the presence of cytoplasmic nucleic acids are the pattern recognition receptors (PRRs), which are more thoroughly studied and well-characterized in most vertebrates. PRRs are innate immunity receptors, defined by their ability to specifically recognize microbes and/or microbial moieties (4). In the presence of invading pathogens, the innate immune response is initiated primarily through the recognition of conserved pathogen-associated molecular patterns (PAMPs) by the PRRs (5). These PRRs serve as a pathogen surveillance system in all eukaryotic organisms, which recognize the conserved molecular pathogen signatures comprised of proteins, lipids and nucleotides (6). The PRRs, therefore, allow the immune system to distinguish self from non-self, while still retaining the capacity to respond effectively during an infection. Ultimately, the recognition of PAMPs by the PRRs trigger the activation of multiple signaling cascades in the host immune cells, including the stimulation of interferons (IFNs) and several other cytokines (6, 7).

PRRs are categorized into three groups depending on their function: (i) soluble bridging PRRs, which facilitate the recognition and elimination of their ligands by phagocytes, (ii) endocytic PRRs, which mediate the recognition and internalization of microbes and/or microbial moieties, and (iii) signaling PRRs, which are involved in cell activation in response to a diverse range of microbial moieties (4, 8). Signaling PRRs are functionally very distinct from the other groups of PRR and are further sub-categorized into three different groups, namely: (i) toll-like receptors (TLRs); (ii) nucleotide oligomerization domain-like receptors (NLRs) and (iii) retinoic acid-inducible gene-I (RIG-1)-like receptors (RLRs) (7, 9–12).

RLRs belong to DExD/H box RNA helicases that are known to be the core cytosolic receptors involved in the recognition of viral RNAs. In mammals, three members in the RLR family have been observed, retinoic acid-inducible gene 1 (RIG-1 or DEAD box polypeptide 58, DDX58), melanoma differentiation-associated gene 5 (Mda5, interferon induced with helicase domain 1, IFIH1, or Helicard), and laboratory of genetics and physiology 2 (LGP2 or DExH box polypeptide 58, DHX58) (13) The RLRs observed in mammals are found to be conservatively present in teleost fish. In fact, all three members, RIG-1, Mda5 and LGP2, have been identified in a range of fish species (14).

The recent advances made in the field of fish immunology over the past few decades, specifically on the knowledge of RLRs in teleost fish, has led the way for to a better understanding of the fish immune system, as well as the diversity and evolution of antiviral immunity in vertebrates. Thus, in this review, we focus on the recent discoveries in relation to PRRs, focusing on Mda5 in particular, which had been identified in several fish species, including model fish species such as zebrafish (*Danio rerio*), and some economically important fish species such as rainbow trout (*Oncorhynchus mykiss*) and Japanese flounder (*Paralichthys olivaceus*).

## General Structure of Mda5 and Its ORTHOLOGS in Fish

**Figure 1:**
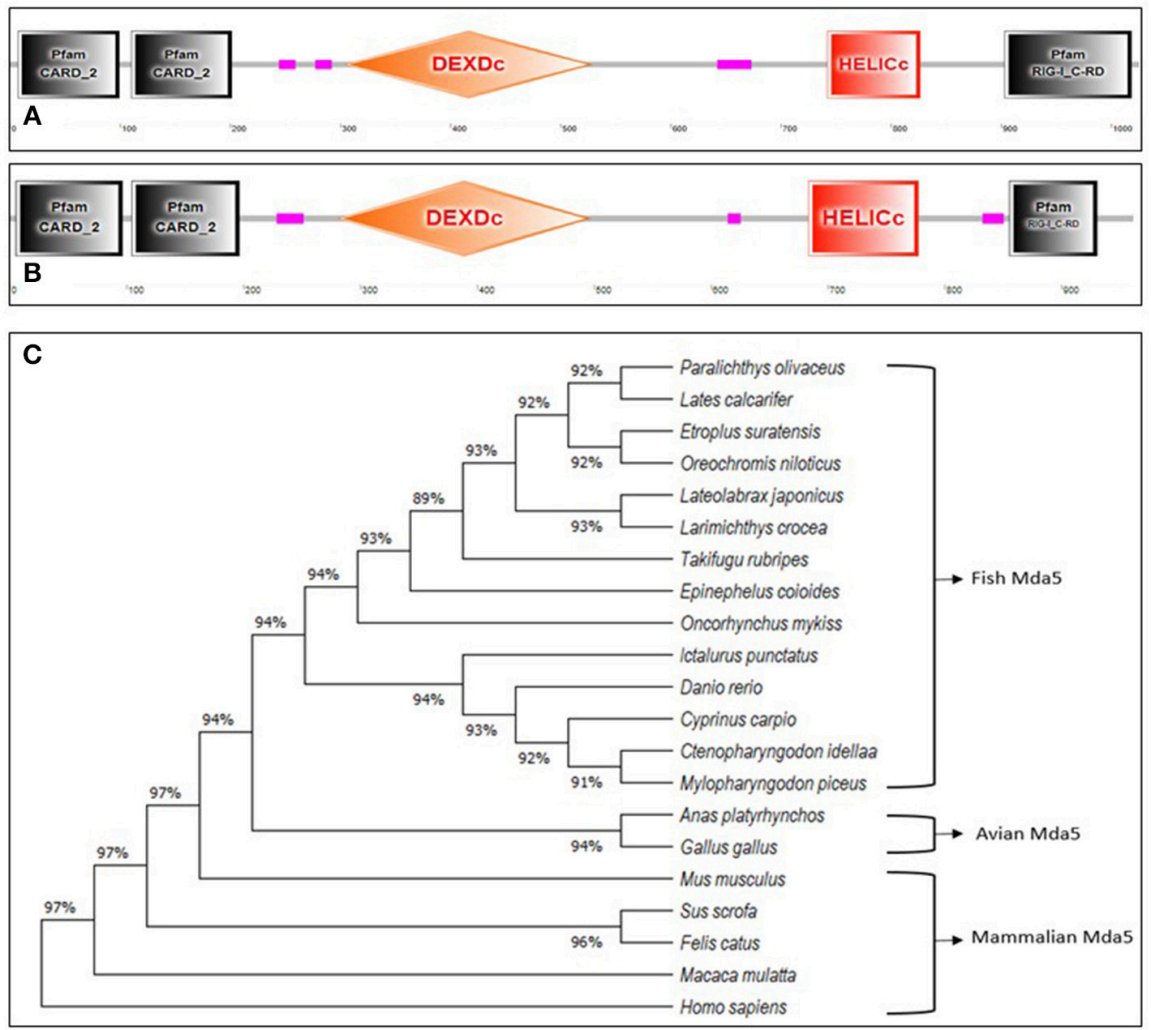
(A,B).Schematic representation of the domain topology of Mda5. The diagram shows two CARD domains, DEXDc, HELICc, and RD domain as predicted by the Simple Modular Architecture Research Tool (SMART) based on the sequence homology. The **(A)** human Mda5 (Accession No. AAG34368.1) and **(B)** grass carp Mda5 (Accession No. AFC88291.1) amino acid sequence were used as templates for the analysis. (The pink boxes signify regions of low compositional complexity and with no features that could be detected) **(C)**. Phylogenetic tree analysis of Mda5. The relationship of the deduced amino acid sequences of fish Mda5 was analyzed in comparison with Mda5 from different groups of animals. The phylogenetic history was inferred by using the Maximum Likelihood method and JTT matrix-based model and phylogenetic analyses were conducted in MEGA X. Accession No. *Paralichthys olivaceus* (ADW78349.1)*, Lates calcarifer* (AOV82292.1)*, Etroplus suratensis* (AIP84311.1)*, Oreochromis niloticus* (AUN88445.1*), Lateolabrax japonicus* (AMW90927.1), *Larimichthys crocea* (ANQ31758.1)*, Takifugu rubripes* (XP_011608571.1)*, Epinephelus coioides *(AEX01716.1)*, Oncorhynchus mykiss *(CAZ27715.1)*, Ictalurus punctatus *(AFS34611.1), *Danio rerio *(NP_001295492.1), *Cyprinus carpio* (AIX47136.1), *Ctenopharyngodon idella* (AFC88291.1), *Mylopharyngodon piceus *(ARO77472.1), *Anas platyrhynchos* (AHW98927.1), *Gallus gallus *(BAJ14020.1*), Mus musculus* (NP_082111.2), *Sus scrofa* (AWH63112.1), *Felis catus *(BAX03651.1), *Macaca mulatta *(ABI33114.1*), Homo sapiens* (AAG34368.1).

Mda5, together with RIG-1, are cytoplasmic sensors of dsRNA comprising of four discrete domains; two caspase recruitment domains (CARDs) at the N-terminal region, a DEAD/DEAH box helicase domain (DEXDc), a regulatory domain (RD) and a helicase C-terminal domain (HELICc) (15, 16) as shown in Figures 1A,B. As observed in humans, Mda5 and RIG-1 proteins in fish are closely related proteins, having structural similarities of 23 and 35% amino acid (aa) identity in their N-terminal tandem CARD and C-terminal helicase domains, respectively (17).

Mda5 (and RIG-1) in fish consists of protein domains that are similar to their mammalian counterparts. The first fish ortholog of Mda5 was reported in pufferfish (*Fugu rubripes*) in 2008, through the use of bioinformatic analyses of available whole genome sequences (18). Since the first characterization of Mda5 in fish, studies focusing on the analysis of this important PRR have increased significantly, partly because of the recent advances made in bioinformatics.

The Mda5 gene has now been cloned and characterized for a number of fish species. Differences in the aa length is quite noticeable when comparing the cloned Mda5 among fish species. As shown in Figures 1A,B, this difference can be attributed to the regions of low compositional complexity along the whole sequence (represented as pink boxes in the diagram). To further elucidate this, the six essential domains of Mda5 were analyzed through the use of the Simple Modular Architecture Research Tool (SMART). As predicted by the tool, the domains were found at different position along the Mda5 sequence in different teleost species, moreover, the aa length of the respective domains also differ (Table 1). These subtle differences in the sequence of Mda5 between different teleost species does not appear to interfere with the function of the protein. Though they differ in ORF length and number of aa residues (see Table 2), analysis of their protein residues show that there is a close phylogenetic relationship between the Mda5 from different fish species and they all have a significant similarity with other vertebrate Mda5 (Figure 1C).

**Table 1:** Comparison of Mda5 according to their domain position as predicted by Simple Modular Architecture Research Tool (SMART).

**Teleost species**	^*^CARD1	^*^CARD2	^*^DEXDc	^*^Helic C	^*^RD
*Ctenopharyngodon idella*	5–96	104–196	281–496	638–777	855–930
*Paralichthys olivaceus*	5–97	107–196	286–498	687–781	859–974
*Oncorhynchus mykiss*	6–99	107–199	301–513	708–796	874–988
*Ictalurus punctatus*	2–94	102–194	306–521	713–801	879–993
*Danio rerio*	9–101	109–201	293–508	695–789	867–981
*Etroplus suratensis*	5–96	108–196	275–501	676–770	848–963
*Lateolabrax japonicus*	7–99	109–198	286–509	687–981	859–974
*Epinephelus coioides*	9–101	112–200	281–493	682–776	853–968
*Cyprinus carpio*	5–98	106–198	289–504	691–785	863–977
*Larimichthys crocea*	5–96	105–195	289–514	690–784	862–977
*Mylopharyngodon piceus*	5–96	106–195	281–496	683–777	855–969
*Lates calcarifer*	7–99	113–198	277–500	678–772	849–964
*Oreochromis niloticus*	5–96	106–196	270–497	671–765	843–958

* start aa-end aa.

**Table 2:** Mda5 orthologs in different teleost species.

**Teleost species**	Nucleotides (bp)^*^	**Amino Acid**	**Accession Number**	**References**
*Ctenopharyngodon idella*	2, 885	961	AFC88291.1	(29)
*Paralichthys olivaceus*	2, 967	988	ADW78349.1	(30)
*Oncorhynchus mykiss*	3, 009	1002	CAZ27715.1	(16)
*Ictalurus punctatus*	3, 018	1005	AFS34611.1	(31)
*Danio rerio*				
Mda5a	2, 994	997	NP_001295492.1	(5)
Mda5b	2, 058	685		
*Etroplus suratensis*	2, 937	978	AIP84311.1	(32)
*Lateolabrax japonicus*	2, 964	987	AMW90927.1	(33)
*Epinephelus coioides*	2, 949	982	AEX01716.1	(34)
*Cyprinus carpio*	2, 982	993	AIX47136.1	(2)
*Larimichthys crocea*	2, 976	991	ANQ31758.1	(35)
*Mylopharyngodon piceus*	2, 955	984	ARO77472.1	(36)
*Lates calcarifer*	2, 937	978	AOV82292.1	(37)
*Oreochromis niloticus*	2, 925	974	AUN88445.1	(38)

* *Open Reading Frame (ORF)*.

## Mda5 and Its Involvement in the Innate Immune Response

In 2002, Mda5 was initially discovered as an interferon-inducible putative RNA helicase with double-stranded RNA-dependent ATPase activity and melanoma growth-suppressive properties in human melanoma cells (19), and then in 2004, Mda5 was reported to play a major role in an intracellular signal transduction pathway, resulting in the activation of the IFN-β promoter, and V proteins of paramyxoviruses were shown to interact with Mda5 to block its activity (20). Subsequent studies have indicated that Mda5 is capable of recognizing a viral infection and transmitting a signal by CARD (21, 22). It was also established that Mda5 could sense single stranded RNA with 5' triphosphate and could selectively recognize long dsRNAs (>3 kb) (18, 23), which includes dsRNA replication intermediates of positive-sense RNA viruses, the genome of dsRNA viruses and polyinosinic: polycytidylic acid (poly I:C). The ability of Mda5 to recognize these dsRNA-related molecules induces the secretion of type I IFN, in particular (24). There had been underlying issues regarding the specificity of ligands that Mda5 and RIG-1 recognized, suggesting an overlap in the mechanism of action between these two RLRs. RIG-1 can be activated by diverse positive- and negative-strand RNA viruses including influenza, Rift Valley fever, measles, Ebola, vesicular stomatitis virus (VSV), and hepatitis C viruses (25). The minimal requirement to activate RIG-1 is a blunt-ended base-paired RNA 10–20 bp long with a 5' triphosphate and free mismatches near the blunt end (denoted 5' _ppp_bpRNA, since this could arise from ssRNA with complementary ends or dsRNA) (26–28). It is also reported that much longer dsRNAs (>200 bp), including poly (I:C), which does not necessarily bear a 5'ppp-end or blunt-ended, can also induce IFN via RIG-1 (23). Thus, it is noteworthy to mention that although poly (I:C) (a synthetic dsRNA commonly used to represent a viral ligand in Mda5 and RIG-1 studies) can induce IFN production through Mda5 and RIG-1 activation, these two RLRs are capable of distinguishing this ligand according to the size wherein, Mda5 tends to recognize long poly (I:C) and RIG-1 specifically reacts with short poly (I:C). Although the mechanism of how these RLRs discriminate between differences in length is not yet understood, we think that it has to do with the “uncoiling” of the dsRNAs and how much ATPase activity is involved for the Mda5 or RIG-1 to be activated.

**Figure 2:**
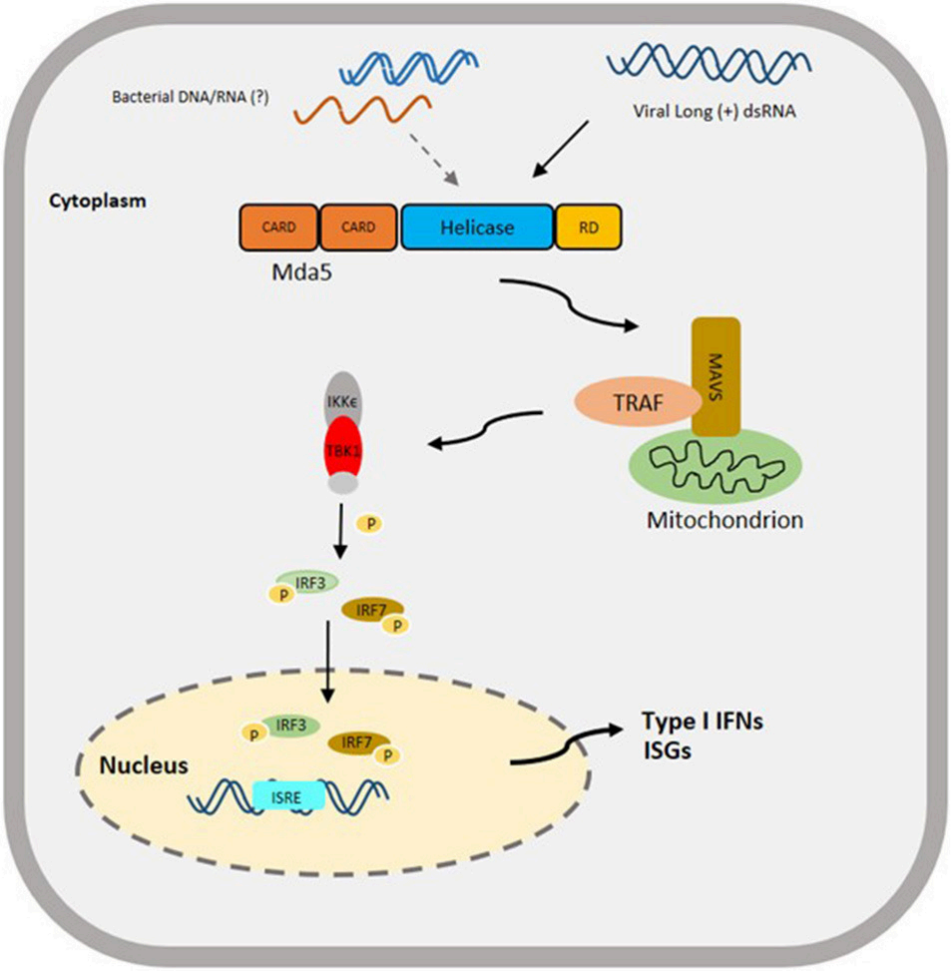
Proposed Schematic diagram of Mda5 signaling pathway in Teleost based on a Mammalian Model. The activation of Mda5 is initiated by the presence of long (+) dsRNA released after viral infection or bacterial nucleic acid, that leads to the phosphorylation of interferon regulatory factor 3 and 7 (IRF3/ IRF7), then to the activation of type I IFN promoter and finally to the expression of type I IFNs and other interferon-stimulated genes (ISGs). CARD, Caspase activation and recruitment domain; RD, regulatory domain; MAVS, mitochondrial-antiviral signaling protein; TRAF, TNF (tumor necrosis factor)-receptor associated factor; IKKϵ, inhibitor of nuclear factor Kappa-B kinase subunit epsilon; TBK1, TANK-binding kinase 1; P, signifies phosphorylation; ISRE, interferon-sensitive response element.

The CARD and helicase domains of the Mda5 are the domains directly involved in the initiation of the signaling pathway, and triggering the innate immune response. The helicase domain binds to dsRNA leading to the activation of the CARD domains. After interacting with PAMPs, the Mda5 CARDs are exposed and they form a complex with the CARD domain of the mitochondrial protein IFN- promoter stimulator-1 [IPS-1, also known as mitochondrial antiviral signaling (MAVS), virus-induced signaling adapter (VISA), and CARDIF], which is located on the outer membrane of the mitochondria (39–42). This is then followed by the recruitment of tumor necrosis factor (TNF)-receptor associated factor 3 (TRAF3) and activation of TRAF family member-associated NF-κB-activator binding kinase-1 (TBK1) and inducible IκB kinase (IKKϵ) (40, 41, 43). The activation of these kinases result in the phosphorylation of interferon regulatory factor 3 and 7 (IRF3/7), the phosphorylated IRF3/7 forms a dimer and translocates to the nucleus to activate the type-I IFN promoter (44). Ultimately, these processes initiate the expression of IFN and pro-inflammatory cytokines. The expression of IFN triggers the release of antiviral effectors, such as IFN-stimulated gene (*isg*) 15, myxovirus resistance gene (*mx*), 2', 5'-oligoadenylate synthetase (OAS)-directed ribonuclease L (RNASEL) pathway and protein kinase R (PKR), which in turn enhances the IFN-mediated antiviral response (45) (Figure 2).

## Mda5 *in vivo* and *in vitro* Expression in Teleost

Synthetic RNAs, IFNs and viruses are known to induce the expression of Mda5 in mammals (21, 46). Studies have shown that fish Mda5 is also capable of responding, both *in vivo* or *in vitro*, to stimulation by synthetic double-stranded RNA (dsRNA), poly(I:C) (30, 32, 35) and to viral infections (29, 33, 34).

The CiMda5 transcripts in grass carp *(Ctenopharyngodon idella)* have been observed to increase in expression after infection with grass carp reovirus (GCRV) *in vivo*, especially in the spleen and liver (29). When expression of rainbow trout Mda5 was examined *in vitro* using rainbow trout gonad (RTG-2) and spleen (RTS-11) cell lines after stimulating the cells with poly(I:C), Mda5 transcripts were observed to increase in both cell lines, but this stimulation was greater in the RTS-11 cells. Intracellular poly(I:C) stimulation also caused a significant increase in Mda5 expression. The expression of Mda5 in RTG-2 cells could also be stimulated using synthesized IFNs (16). Expression of Japanese flounder Mda5 was evaluated *in vitro* using whole kidney leukocytes (KL) and peripheral blood leukocytes (PBL) with poly(I:C) stimulation, and also *in vivo* with viral hemorrhagic septicemia virus (VHSV), with significant up-regulation of Mda5 transcripts noted both *in vitro* and *in vivo* (30).

The studies outlined above have clearly shown that Mda5 is able to be stimulated, both appropriately and efficiently, by synthetic stimulators such as poly(I:C) and viral infections in either fish or in fish cell lines, and have laid the ground work for subsequent studies examining the Mda5 response to viral infections in other fish species. As summarized in Table 3, fish Mda5 can strongly be up-regulated in different teleost species through the use of different stimulants, either with a virus or poly(I:C) as observed in different cell lines and organs.

**Table 3:** Up-regulation of Mda5 by different viral stimulants in other teleost species.

**Fish species**	**Stimulant**	**Experiment**	**Organs/cells observed**	**References**
Channel catfish	Channel catfish virus (CCV)	*in vitro*	Ovarian channel catfish cells	(31)
Zebra fish	spring viraemia of carp virus (SVCV)	*in vitro*	Zebrafish cell line, ZF4	(5)
Green chromide	Poly I:C	*in vivo*	Spleen, intestine, heart, gill, kidney, liver	(32)
Sea perch	Nervous Necrosis virus (NNV) poly I:C Redspotted grouper nervous necrosis virus (RGNNV)	*in vivo in vitro*	Spleen, kidney *Lateolabrax japonicus* brain (LJB) and fry (LJF) cells	(33)
Orange spotted grouper	Poly I:C Singapore grouper iridovirus (SGIV)	*in vivo*	Spleen	(34)
Common carp	Poly I:C	*in vivo*	Spleen, liver, head kidney, foregut, hindgut, gill, skin	(2)
Large yellow croaker	Poly I:C	*in vivo*	Peripheral blood, liver, head kidney, spleen	(35)
Black carp	Poly I:C SVCV grass carp reovirus (GCRV)	*in vitro*	*Mylopharyngodon piceus* fin (MPF) cells	(36)

Mammalian Mda5 is established as a viral PAMP-recognizing PRR of different ssRNA, dsRNA viruses as well as poly(I:C), which is a synthetic analog of dsRNA virus. In the case of teleost Mda5, this PRR has been implicated in the stimulation of the immune response against viral antigens, probably by serving as a sensor. However, in the study performed by Ohtani et al. (30), in which they used a synthetic bacterial analog, Lipopolysaccharide (LPS), representing stimulation by Gram-negative bacteria, their results showed up-regulated Mda5 expression after LPS stimulation, suggesting that Mda5 might not be involved exclusively in recognizing viral PAMPS, but they are also capable of indirectly distinguishing bacterial PAMPs. Several studies concurrently showed that LPS or bacterial challenge resulted in up-regulation of fish Mda5, such as in channel catfish (*Ictalurus punctatus*) challenged with *Edwardsiella ictaluri* (31), in common carp (*Cyprinus carpio*) after *Aeromonas hydrophila* challenge (2) and in black carp (*Mylopharyngodon piceus*) MPF cells after LPS stimulation (36).

The most recent studies on fish Mda5 further verify that this PRR is indeed not only involved in virus detection, but also has the ability to initiate the RIG-1/Mda5 pathway during bacterial infection. The expression level of Asian seabass Mda5, AsMda5, in response to bacteria was elucidated by infecting juvenile fish with either *Vibrio alginolyticus* (Gram-negative bacterium) or *Staphylococcus aureus* (Gram-positive). When Sahul India seabass kidney (SISK) cell line was exposed to LPS, a sustained level of AsMda5 up-regulation was obtained several hours after stimulation, but the levels of expression obtained were not as high as those seen in fish stimulated with LPS *in vivo*. Poly(I:C)-injected fish, on the other hand, produced much higher levels of AsMda5 expression than fish injected with bacterial LPS (37). In tilapia (*Oreochromis niloticus*), a *Streptococcus agalactiae* (Gram-positive bacterium) infection caused an increase in OnMda5 transcript levels in the intestine, kidney, gills and blood at different time points (38). Together, the results highlighted above for the various fish species indicate that fish Mda5 is not only involved in antiviral immune responses, but also bacterial-triggered immune responses, although the mode of action of Mda5 stimulation by bacteria has yet to be determined.

Bacterial ligands are recognized by a different group of PRRs, nucleotide-binding oligomerization domain (NOD)-like receptors (NLRs) and some toll-like receptors (TLRs), which had been widely observed among vertebrates. NLRs can cooperate with TLRs and regulate inflammatory and apoptotic responses. Different mammalian TLR families had been elucidated and most of them have also been found in teleost fish including two additional fish-specific TLR family members (47, 48). Although these fish orthologs have already been demonstrated in different species, the role of these TLRs in the recognition of ligands from bacteria is now the focus of intense studies. NLRs, specifically, NOD1 and NOD2, recognize peptidoglycan components common to both Gram-positive and Gram-negative bacteria. Both proteins drive activation of mitogen-activated protein kinase (MAPK) and nuclear factor κ-light chain-enhancer of activated B cells (NF-κB) pathways, leading to pro-inflammatory cytokine production (49, 50). There has not been a direct link between these receptors, except for the fact that when these receptors are activated, adaptor proteins (i.e., MyD88, MAVS) trigger a downstream cascade leading to the release of inflammatory genes needed in the fight against pathogens (51). The studies mentioned above, on the overexpression of Mda5 after bacterial challenge, do not provide any additional information on the mechanism involved or if it is definite that Mda5 recognized these bacterial ligands. We, therefore, hypothesize that maybe the presence of excess number of bacterial nucleic acids (i.e., small RNAs) which were indirectly sensed by Mda5 led to the observed overexpression of this PRR. We also believe that if they had included RIG-1 in their analysis, an up-regulation of RIG-1 could also have been observed, but the study focused more on Mda5, and they did not delve into TLR/NLR markers. The limiting factor of previous studies is that lack of expression of any TLR/NLR as a comparison, as this would further explain if these two totally different group of PRRs perform overlapping immune response mechanisms in fish.

## Antiviral and Antibacterial Functions of Mda5

The essential role of Mda5 in antiviral responses had been suggested by the existence of paramyxovirus proteins. The highly conserved cysteine-rich C-terminal domain of the V proteins of a wide variety of paramyxoviruses binds to Mda5 products. As shown from reporter assays, Mda5 stimulates the basal activity of the IFN-β promoter, and over-expression of Mda5 enhances the activation of IFN-β in response to intracellular dsRNA. It was also shown that Mda5 can activate both NF-κB and IRF-3, suggesting that Mda5 plays a pivotal role in the upstream activation of these transcription factors in response to dsRNA, however, these activities were repressed by co-expression of the V proteins (20). The V protein of the Sendai virus, Hendra virus, simian virus 5, human parainfluenza virus 2, and mumps virus selectively abrogates Mda5 function, highlighting the ingenious mechanisms of initiating antiviral immune responses and the action of virus-encoded inhibitors (17, 20).

It was demonstrated using knockout mice that Mda5 plays a crucial role in type I IFN responses by dendritic cells (DCs) and macrophages, when the mice were stimulated with poly(I:C). Specifically, Mda5-deficient mice showed abrogated production of IFN-α and IFN-β in bone marrow-derived DCs and macrophages, and that this PRR is functionally dominant over TLR3 for type I IFN responses to poly(I:C) *in vitro* and *in vivo*. Furthermore, mice without Mda5 activity succumbed sooner to infection by encephalomyocarditis virus (ECMV), confirming the essential role that Mda5 has in the host's resistance to ECMV *in vivo* (21).

In comparison to mammals, innate immunity in teleost fish is poorly understood. It is therefore important to establish how PAMPs recognize PRRs in fish. Earlier reports on fish PRRs, especially Mda5, focused on molecular characterization instead of function, and it is only in the past decade that studies have focused on the role of these molecules in the innate immunity of fish.

When the effect of grass carp Mda5 (CiMda5) on the production of various IFNs in *C. idella* kidney (CIK) cells infected with grass carp reovirus (GCRV) was examined, CiMda5 was shown to induce an extensive IFN response in the infected cells by facilitating total phosphorylation of IRF3 and IRF7, enhancing the heterodimerization of IRF3 and IRF7 and the homodimerization of IRF7. The homodimer IRF7 broadly induces the production of IFN-1 in response to GCRV infection, suggesting that CiMda5 has a crucial role in the cytosolic pathway for the induction of IFN genes in response to GCRV (52). Meanwhile, over-expression of Mda5 in Hirame natural embryo (HINAE) cells resulted in a decreased cytopathic effect in cells infected with VHSV, hirame rhabdovirus (HIRRV) and infectious pancreatic necrosis virus (IPNV). The observed reduction in VHSV titers indicate that Japanese flounder Mda5 inhibits the replication of ssRNA viruses (VHSV and HIRRV) as well as dsRNA viruses (IPNV). Moreover, the expression level of type I IFN, Mx and ISG15 genes in Mda5-overexpressing HINAE cells, infected with VHSV, was significantly higher than in non-infected cells. These results demonstrate the ability of Japanese flounder Mda5 to enhance the antiviral activity of the fish, mediated by the activation of type I IFN and IFN-stimulated genes (30). In the zebrafish cell line ZF4, overexpression of the two splice variants of Mda5 (Mda5a and Mda5b) significantly induced type I interferon promoter activity and promoted protection against SVCV infection in transfected cells (5). In another zebrafish experiment, overexpression of Mda5 in zebrafish liver (ZFL) cells had a 2.5 × 10^6^ -fold reduction in viral burden after infected with snakehead rhabdovirus (SHRV) demonstrating that Mda5 overexpression increases resistance to SHRV (1). Overexpression of orange spotted grouper (*Epinephelus coioides*) Mda5 triggered an increase in the expression levels in IFN and IFN-stimulated response element (ISRE) promoter in a dose-dependent manner (400 and 800 ng ml^−1^) and also enhanced the expression of IRF3, IRF7, and TRAF6 (TNF receptor-associated factor 6) and some pro-inflammatory factors including, tumor necrosis factor (TNF-α), interleukin 6 (IL-6) and IL-8 at different time points during SGIV and RGNNV infection (34).

The role of Mda5 in the fish innate immunity by the induction of IFN-mediated immune response after viral infection has been well-elucidated. In addition, studies have demonstrated that Mda5 can also be triggered by bacterial stimulation. However, the studies carried out on fish, showing the ability of bacterial stimulants in up-regulating Mda5 expression, did not specifically discuss the mechanism behind this occurrence, but it has also been observed in other animals. In fact, there are studies in mammals which show that the RIG-1/Mda5 pathway, thought primarily to detect viruses, is also involved in the innate immune response to intracellular bacteria e.g., *Legionella pneumophila*, a Gram-negative bacterium (53) and *Listeria monocytogenes*, a Gram-positive bacterium (54). *Listeria monocytogenes* releases nucleic acids during the infection that are recognized by the cytosolic sensors RIG-1, Mda5, and stimulator of interferon genes (STING), thus resulting in the expression of IFN-β and an inflammasome response (54).

The involvement of Mda5 in the innate immune response in fish is limited to the results presented above, and it has not been confirmed that Mda5 acts as a receptor for viral and/or bacterial ligands. Instead, we can only generalize that the overexpression of Mda5 can lead to the protection of fish, therefore, investigating the “ligand-receptor” interaction of fish Mda5 could give us a better insight if this PRR has an equivalent function to that observed in higher vertebrates.

## Interaction of Mda5 With LGP2

Our understanding of fish immunology had increased greatly over the past few decades, including the discovery of orthologous genes for mammalian RIG-1, Mda5, and LGP2 (14). The functional characteristics of these RLRs have been investigated in a range of teleost species, including model fish species such as zebrafish, and these genes appear to have similar functions to those present in mammals [5, 53].

All three RLRs share homologous core structural domains, including a DExD/H box helicase domain, a helicase C-terminal domain and a C-terminal domain (CTD), however, while RIG-1 and Mda5 have CARDs, LGP2 lacks them (55), and this absence of CARDs in LGP2 makes it unable to induce signaling alone. This is consistent with the inability of LGP2 to intrinsically activate the IFN-β promoter in transient overexpression experiments (56). Thus, it is difficult to determine its exact role in RLR-mediated signaling (57), and until now, the function of LGP2 in antiviral signaling has been controversial.

LGP2 has been identified as a negative regulator of IFN response, for example when triggered by Sendai virus, Newcastle disease virus (17, 56, 58, 59) or poly (I:C) (58). However, mice studies have shown that LGP2 can have either a positive or a negative role in IFN induction (60). The action of LGP2 is believed to synergize with that of Mda5, but not RIG-1, to boost IFN signaling at low levels of LGP2 expression. On the other hand, at higher levels of LGP2 expression, it acts as an inhibitor of RIG-1 and Mda5 signaling (61–63). In recent studies, mammalian LGP2 has been shown to be involved in Mda5 filament formation and Mda5-mediated viral RNA recognition (63, 64). Other studies have confirmed that LGP2 synergizes with Mda5: (1) to elevate IFN- transcription *in vivo*, for example during an encephalomyocarditis virus infection or poly (I:C) stimulation (62); (2) to facilitate viral RNA recognition through its ATPase domain (65) and (3) to sense Sendai virus infection for IFN-1 induction along with the loss of RIG-1, as determined in Chinese tree shrew (66).

Some reports have shown that teleost LGP2 is a negative regulator of antiviral immunity when overexpressed *in vitro*. For example, overexpression of crucian carp (*Carassius carassius*) LGP2 reduced the activity of IFN promoters, mediated by RIG-1 and Mda5 (67), and down-regulation of antiviral immune genes like Mda5, was also observed in cells overexpressing the grouper LGP2 (68). In zebrafish, LGP2 negatively regulates the IFN response mediated by poly (I:C) by blocking some of the important signaling factors, including RIG-1 and Mda5, but not IRF3/7 (69). It also appears that the antithetical function of LGP2 in antiviral immunity depends on LGP2 expression levels, similar to that is observed with mammalian LGP2. Zebrafish LGP2 functions as a positive regulator of IFN signaling during the early phase of virus infection; during this time RIG-1 and Mda5 are expressed at low levels, while during latter phases of the infection, LGP2 adopts a negative role. However, the maximum stimulatory effect of zebrafish LGP2 is lower than levels of Mda5 and RIG-1 expression (70). These results have also been corroborated for grass carp LGP2. During the resting state and early phase of grass carp reovirus (GCRV) infection, synthesis and phosphorylation of IRF3/7, and mRNA levels and promoter activities of IFNs and NF-κBs are inhibited, at a time when grass carp LGP2 is overexpressed. Luciferase assay have shown that grass carp LGP2 binds to RIG-1 and Mda5 with diverse domain preference, and this binding is independent of the GCRV infection. Another interesting result showed that grass carp LGP2 inhibits K63- and K48-linked RIG-1 and Mda5 ubiquitination, resulting in suppression of protein degradation. These results indicate that LGP2 has a role as a suppressor in RLR signaling pathways, which is important in maintaining cellular homeostasis during the resting state and early phase of GCRV infections (71).

**Figure 3:**
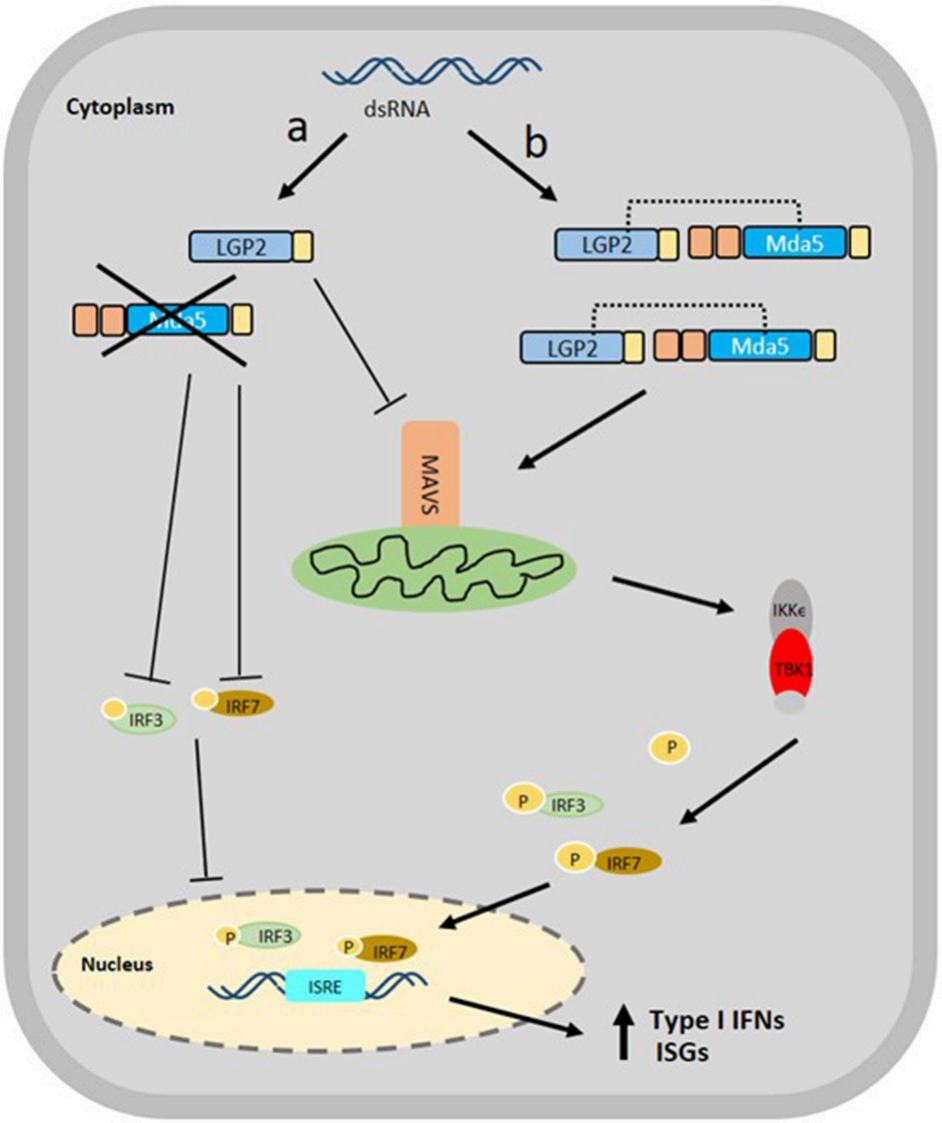
Proposed Schematic diagram of the interaction of Mda5 with LGP2 in the RLR signaling pathway. The presence of dsRNA in the cytoplasm is recognized by both RLRs, however, the signaling mechanism can follow two different paths: (a) the LGP2 acts as a *negative regulator* of type I IFN response that causes inhibition of the Mda5 and suppression of the expression of MAVS, IRF3, and IRF7 and (b) the LGP2 functions as a *positive regulator* in which LGP2 nucleates MDA5 filaments by binding dsRNA termini extending the MDA5 filaments. The LGP2 stabilizes the formation of these shorter filaments for the proper attachment to MAVS, thereby, causing more efficient and up-regulated expression of IRF3 and IRF7, leading ultimately, to the up-regulation of secreted type I IFNs and ISGs.

On the other hand, black carp LGP2 (bcLGP2) was clearly shown to have a synergistic effect with bcMda5 using reporter assays, in which, both the induction of zebrafish IFN3 and fathead minnow IFN (eIFN), mediated by bcMda5 and bcLGP2, were much higher than that obtained by bcMda5 alone, and was higher than the combined effect of bcMDa5 or bcLGP2 alone. The synergistic function between bcLGP2 and bcMda5 reflects bcLGP2 effect on the antiviral activity of the host. Epithelioma papulosum cyprini (EPC) cells, overexpressing both bcLGP2 and bcMda5, showed a decrease in CPE development and viral titer during infection with GCRV or SVCV, in contrast with cells expressing either bcMda5 or bcLGP2 alone (36). In a study focusing on rainbow trout Mda5 and LGP2, it was evident that both RLRs are capable of binding to poly (I:C), triggering IFN production. Also, Mda5 expression is not affected by the overexpression of LGP2 in transfected cells, and *vice versa*, implying that these RLRs function in parallel as positive regulators for IFN production (16). Although these results help to substantiate the synergy between LGP2 and Mda5, the mechanism behind their interaction remains unclear. A proposed mechanism of action showing the antithetical role of LGP2 with Mda5 is suggested in Figure 3.

## Does Mda5 Have a Major Role in Acanthopterygians?

The three RLRs, RIG-1, Mda5, and LGP2, are represented in a number of teleost species, however, one intriguing discovery is the absence of RIG-1 in some members of Acanthopterygii. Presently, RIG-1 has only been identified in crucian carp (67), grass carp (72), common carp (73), zebrafish (74, 75), channel catfish (31), Atlantic salmon and EPC (70). Despite efforts to identify RIG-1 fish orthologs, it has not been possible to identify RIG-1 in Japanese pufferfish (*Takifugu rubripes*) and green spotted puffer fish (*Tetraodon nigroviridis*) (15, 18), medaka (*Oryzias latipes*), and three-spined stickleback (*Gasterosteus aculeatus*) (15), and rainbow trout (16), gilt-head sea bream (*Sparus aurata*) and European bass (*Dicentrarchus labrax*) (76). In addition, *in silico* data mining of Japanese flounder, Nile tilapia and orange-spotted grouper genomes also showed the absence of RIG-1 in the genome of these teleost species (77).

Orthologs of Mda5 and LGP2 seem to be common to all teleost families, unlike RIG-1, which is only found in more primitive fish species, such as those in classes Ostariophysi, Protacanthopterygii, and Paracanthopterygii (77). It is believed that Mda5 might have emerged before RIG-1 and that the domain arrangements of the genes evolved independently by domain grafting rather than a simple gene duplication event (18).

Furthermore, the presence of the three RLRs in very ancient fish, Sarcopterygii, imply the loss of RIG-1 after the divergence of the Acanthopterygii from the Paracanthopterygii (77). Interestingly, most of the fish species reared for aquaculture do not possess the RIG-1 ortholog, and all of them belong to the superorder Acanthopterygii.

With the absence of RIG-1 in some of the Acanthopterygians, their RLRs have possibly evolved differently, wherein their Mda5 as well as LGP2 perform most of the pivotal role in antiviral sensing. Knowing that LGP2 and Mda5 have the capability to work synergistically, it is important to help establish if these two RLRs function in place of RIG-1 in these fish species, and if not, what is the equivalent gene that performs the role of RIG-1? For instance, in chicken, another organism that lacks RIG-1, studies found that chicken Mda5 compensates for the lack of RIG-1 by preferentially sensing shorter dsRNA synthetic poly (I:C) instead of long dsRNAs (78). Another example of mammal that does not have RIG-1 is the Chinese tree shrew. It was revealed that the loss of RIG-1 brought positive selection signals to tree shrew Mda5(tMda5) and LGP2(tLGP2). Data showed that tMda5 alone or tMda5/tLGP2 could replace RIG-1 as a sensor for RNA viruses that trigger IFN production (66). It is believed that this replacement is enhanced due to the interaction of tMda5 with tMITA (Mediator of IRF3 activation), which interacts with RIG-1 resulting in a cascade of antiviral signaling (79). This information tells us that even in the absence of RIG-1, the innate immune system has a way of compensating for the loss of some molecules by relying on other functional molecules, probably homologs, although in fish, the compensatory effect of Mda5 in teleost lacking RIG-1 has not yet been verified, and further investigation is essential to establish this. A study was performed on the RLRs, Mda5, and LGP2 of rainbow trout (*Oncorhynchus mykiss*), focusing on the parallel function of these two RLRs that synergistically increase in the production of IFNs (16), but whether this was in compensation for the lack of RIG-1 in this fish was not specifically discussed.

## Concluding Remarks and Future Perspectives

The teleost immune system may not be as elaborate as that of its mammalian counterparts, but they have an intricate innate immune system that is on a par with the complex immune system present in mammals. Since the aquatic environment in which fish live is very different to that of mammals, where they are in close contact with pathogens, it is important that their innate immune system offers a first line of defense against invading pathogens. The PRRs in teleosts, similar to that present in mammals, are capable of sensing pathogens and inducing antiviral and/or antibacterial responses. Knowing the role that Mda5 plays during an infection will help give a clearer insight of how the teleost immune system works. Mda5, together with other RLRs, are able to sense pathogens and, in turn, activate downstream processes in the fish's immune response, ultimately, preventing them from succumbing to the infection.

Although, Mda5 was described for several different teleost species in this review, the mechanism of action of Mda5 still needs further elucidation. As discussed, Mda5 expression directs the recruitment of the downstream adaptor MAVS from the mitochondria, then associates with signaling molecules like TBKI, TRAF3, and MITA, which in turn facilitate the activation and phosphorylation of IRF3 and IRF7, leading to their translocation into the nucleus for the induction of type I IFNs and ISGs. These downstream molecules have been identified in various teleost species as a result of stimulation with poly(I:C) and LPS, as well as viral and bacterial infections, however, the extent to which Mda5 regulates the whole process of initiating innate immunity in fish has yet to be established and whether Mda5 works in cooperation with other PRRs thereby suggesting a network of immune molecules instead of a single, linear pathway.

Further studies are needed to establish how these PRRs function within the teleost immune system, for example:

1. RLRs that recognize viral ligands include RIG-1, Mda5, and LGP2. RIG-1 and Mda5 recognize distinct viral dsRNA. LGP2, on the other hand, recognizes the same viral ligand as RIG-1. The molecular signaling mechanisms of RIG-1 and Mda5 are known to share some common features and LGP2 has been found to be a co-stimulatory molecule for Mda5. It appears that these RLRs have overlapping mechanism of action upon virus invasion. It is therefore important to further characterize their function to be able to differentiate them from one another, especially with respect to the downstream signaling cascade they initiate during an antiviral response.
2. Mda5 shows positive up-regulation in the presence of bacterial ligands, such as LPS and bacteria (both Gram positive and negative strains) suggesting that its activity is not limited only to viral, but also bacterial sensing. Since most of the studies relating to teleost Mda5 have focused on its antiviral response, further studies investigating the role of Mda5 in sensing bacterial PAMPs or its ability to interact with other bacterial PAMP-sensing PRRs is warranted. Bacterial infection in teleost fish showed overexpression of Mda5, but the studies mentioned in this review did not address the specific immune genes involved. Knowing this that would help explain the mechanisms of protection elicited during bacterial infections.
3. The absence of RIG-1 in Acanthopterygians poses the question whether this group of teleosts has evolved a different gene that performs viral sensing, especially, for short dsRNAs. Studies examining the activity of Mda5 (and LGP2) in this group of teleosts are needed to establish if these two RLRs are able to sense all types of viral RNAs in fish lacking RIG-1.

In summary, the fish innate immune system is not as simple as often described, and although it is less complex than the mammalian immune system, it has evolved many similar defense mechanisms that are present in terrestrial organisms. Future elucidation of the regulatory mechanism of Mda5 during pathogen infection is required for a more comprehensive understanding of the role of this and other PRRs in the immune response of fish.

## Author Contributions

JL designed and wrote the draft. KT contributed by reading and giving several discussion. TJ organized and finalized the paper.

### Conflict of Interest Statement

The authors declare that the research was conducted in the absence of any commercial or financial relationships that could be construed as a potential conflict of interest.
